# Supplementary material for: A Comprehensive Analysis of Physiologic and Hormone Basis for the Difference in Room-Temperature Storability between ‘Shixia’ and ‘Luosanmu’ Longan Fruits
Source: Plants (Basel). 2022 Sep 25;11(19):2503. doi: 10.3390/plants11192503 (PMC9572663; doi:10.3390/plants11192503)

A Comprehensive Analysis of Physiologic and Hormone Basis for the Difference in Room-Temperature Storability between 'Shixia' and 'Luosanmu' Longan Fruits

Libing Long [1,†], Tingting Lai [1,†], Dongmei Han [2,†], Xiaolan Lin [1], Jianhang Xu [1], Difa Zhu [1], Xiaomeng Guo [1], Yuqiong Lin [1], Fengyi Pan [1], Yihang Wang [1], Ziying Lai [1], Xinxin Du [1], Di Fang [1], Liang Shuai [3], Zhenxian Wu [1,*] and Tao Luo [1,*]

[1] College of Horticulture, South China Agricultural University/Guangdong Provincial Key Laboratory of Postharvest Science of Fruits and Vegetables/Engineering Research Center of Southern Horticultural Products Preservation, Ministry of Education, Guangzhou 510642, China
[2] Institute of Fruit Tree Research, Guangdong Academy of Agricultural Sciences/Key Laboratory of South Subtropical Fruit Biology and Genetic Resource Utilization, Ministry of Agriculture, Guangzhou 510640, China
[3] College of Food and Biological Engineering/Institute of Food Science and Engineering Technology, Hezhou University, Hezhou 542899, China
* Correspondence: zhenxwu@scau.edu.cn (Z.W.); luotao0502@scau.edu.cn (T.L.)
† These authors contributed equally to this work.

Table S1 Specific primers used for qRT-PCR of pericarp samples.

Gene name	Annotation & Genome_ID	Primer (F: 5’-3’ or R: 5’-3’)	Product (bp)
Actin	actin-101;	F: ACTGCTGAACGGGAAATCG	172
	Dil.11g013910.1	R: CTTCTGGACAACGGAACCTCT	
PPO	chloroplastic polyphenol oxidase;	F: AAAAGCACCGTGGTTCCG	147
	Dil.07g017280.1	R: CGCCTGGTCCTCATCGTTAAT	
POD	peroxidase 4;	F: TCGGTGAGAGGGTTTGAGGT	173
	Dil.03g012870.1	R: CTGGCTGACTTTGAATCCCTT	
CAT	Catalase isozyme 1;	F: GCAGTGGAAAGCGTGAAAAG	184
	Dil.09g001430.1	R: GCCTGAGACCAGTATGAGACCC	
APX	L-ascorbate peroxidase 2,	F: TGCCTTATCTGGCGGTCACAC	231
	cytosolic; Dil.14g006840.1	R: TCAGCAAAGAAAGCATCCTCATC	
GR	chloroplastic glutathione reductase;	F: TCCTGACATTCCTGGCAGTGAGTAT	106
	Dil.04g006420.1	R: CAACGCAATATAACCACCACCAACTATC	
GPX	chloroplastic glutathione	F: AGTTTACCAGTTTCTGAAGTCGAGC	126
	peroxidase 1; Dil.01g007720.1	R: GGTGACGTTGTTGGCGGAT	
NCED1	9-cis-epoxycarotenoid	F: TCACTTATTTAGCCCTTGCGG	210
	dioxygenase; Dil.02g010420.1	R: GCTGCGATTTCCACTCCTTC	
ABA2	xanthoxin dehydrogenase 2;	F: TCAAAGATTAGTTGGGAAAG	217
	Dil.10g020650.1	R: AACAGCAGAGCGAACATC	
AAO3	abscisic-aldehyde oxidase-like;	F: GCTAACTCGTTGTGCTGGTC	128
	Dil.13g015420.1	R: CTGTTAGTATGGGAAGCTCTAG	
ABF	Abscisic acid-insensitive 5-like;	F: GGAAAGAGCAATGGGGATACA	121
	Dil.09g011430.1	R: TTCTTCTCTGCCTCCTCTCAAC	
LOX2	linoleate 13S-lipoxygenase 3- 1,	F: CTAACCGTCCACCCCTAAT	146
	chloroplastic; Dil.03g001200.1	R: TGTGTCAACCACAGCCATGT	
JAZ	protein TIFY 10B;	F: CGGGCAGGTCATCGTGTTC	217
	Dil.01g002090.1	R: GCATTTCGGTTCAGTTGGGTT	
PAL1	phenylalanine ammonia lyase;	F: TGGAAGCCATCGCCAAGTT	103
	Dil.04g021490.1	R: GCAATGTAGGACAGTGGGACC	
PR-1	pathogenesis-related protein PR1-1;	F: GCAAAAACCTACGCCAACC	137
	Dil.11g016370.1	R: GGCTTCTCATCCACCCACA	
NPR3	Regulatory protein NPR3;	F: GGTTCTCAGCCTTTGTTTG	281
	Dil.07g000580.1	R: TTGGATTCCTCCTCATTTC	

Table S2 Specific primers used for qRT-PCR of aril samples.

Gene name	Annotation & Genome_ID	Primer (F: 5’-3’ or R: 5’-3’)	Product (bp)
Actin	actin-101;	F: ACTGCTGAACGGGAAATCG	172
	Dil.11g013910.1	R: CTTCTGGACAACGGAACCTCT	
POD	peroxidase 4;	F: TCGGTGAGAGGGTTTGAGGT	173
	Dil.03g012870.1	R: CTGGCTGACTTTGAATCCCTT	
CAT	Catalase isozyme 1;	F: GCAGTGGAAAGCGTGAAAAG	184
	Dil.09g001430.1	R: GCCTGAGACCAGTATGAGACCC	
APX	L-ascorbate peroxidase 2,	F: TGCCTTATCTGGCGGTCACAC	231
	cytosolic; Dil.14g006840.1	R: TCAGCAAAGAAAGCATCCTCATC	
GR	chloroplastic glutathione reductase;	F: TCCTGACATTCCTGGCAGTGAGTAT	106
	Dil.04g006420.1	R: CAACGCAATATAACCACCACCAACTATC	
GPX	chloroplastic glutathione	F: AGTTTACCAGTTTCTGAAGTCGAGC	126
	peroxidase 1; Dil.01g007720.1	R: GGTGACGTTGTTGGCGGAT	
PE	pectinesterase 1;	F: TGAAGGTTTGCGGGACAGT	194
	Dil.14g014020.1	R: GGTGGCGTTCAAGTTTAGTTCT	
PG	Polygalacturonase;	F: ATGGTAAGGCAACCGAAACTG	210
	Dil.10g001250.1	R: CCGAACCCAAGGTATGAAAAG	
CX	endo-(1,4)-*β*-D-glucanase;	F: ACCAAACTTTCCAAAGAGAATCCAC	150
	Dil.09g022610.1	R: CCGACAATGGCTCCGACTAAG	
β-Gal	β-galactosidase 16;	F: CAAACCGACTGGCAACCTAT	265
	Dil.09g014680.1	R: TCCAACAGCGTAACTATCACAATC	
NCED	carotenoid cleavage dioxygenase 4;	F: CTTGCCTCGTGGTCCTTA	107
	Dil.14g000520.1	R: TGTTGGTCTTGACATAGCGG	
ABA2	xanthoxin dehydrogenase 2;	F: ACAGGATTGGCTTTGGCTC	116
	Dil.10g020650.1	R: TCAACCGTCAACTCCACTCC	
AAO3	abscisic-aldehyde oxidase-like;	F: GCTAACTCGTTGTGCTGGTC	128
	Dil.13g015420.1	R: CTGTTAGTATGGGAAGCTCTAG	
ABF	Abscisic acid-insensitive 5-like;	F: GGAAAGAGCAATGGGGATACA	121
	Dil.09g011430.1	R: TTCTTCTCTGCCTCCTCTCAAC	
LOX2	linoleate 13S-lipoxygenase 2- 1,	F: GTTGGTGCGATTTCGGTGG	101
	chloroplastic; Dil.13g013000.1	R: GCACAGTTTATGCTAACGGGACC	
JAZ	protein TIFY 10B;	F: CGGGCAGGTCATCGTGTTC	217
	Dil.01g002090.1	R: GCATTTCGGTTCAGTTGGGTT	
PAL1	phenylalanine ammonia-lyase;	F: GAGCAGCACAACCAGGATG	237
	Dil.14g007600.1	R: GCAGAATCTTGATGGGTGTAGTT	
PR-1	pathogenesis-related protein PR1-1;	F: GCAAAAACCTACGCCAACC	137
	Dil.11g016370.1	R: GGCTTCTCATCCACCCACA	
NPR3	Regulatory protein NPR3;	F: CGACGCTATTTCCCCCATT	195
	Dil.07g000580.1	R: TGCTGAGGAAGACAACCCG	

Table S3 Prediction analysis of cis-elements related to hormone of promoter sequence in genes used in qRT-PCR.

**Gene Name**	**cis-elements related to hormone (Position: bp to ATG, Strand)**			
**Genome_ID**	**ABA**	**MeJA**	**SA**	**Others (**GA; Auxin**)**
PPO, Dil.07g017280.1	ABRE(1712, +; 1677, +; 986, -; 161, +)	CGTCA(1779, +); TGACG(1779, -)	TCA(655, -)	GA: P-box(1957, -), TATC-box(1566, -)
POD, Dil.03g012870.1	ABRE(362, +; 232, +)	TGACG(1686, +); CGTCA(350, +)	TCA(294, -)	Auxin: AuxRR(437, -); GA: P-box(689, -)
CAT, Dil.09g001430.1	ABRE(941,+; 153, -)	TGACG(1676, +)	----	Auxin: AuxRR(242, +); GA: GARE(1807, +)
APX, Dil.14g006840.1	ABRE(1687, -; 465, -; 459, -; 366, -)	TGACG(1676, +)	TCA(711, +)	GA: GARE(1432, -)
GR, Dil.04g006420.1	ABRE(1376, -; 142, -; 141, +)	TGACG(1117, -; 342, -; 317, +; 135, +)	----	Auxin: AuxRR(1082, +)
GPX, Dil.01g007720.1	ABRE(1321, +; 133, -; 132, +; 82, -)	TGACG(1916, -)	----	----
PE, Dil.14g014020.1	ABRE(1908, +; 1906, +; 1905, +; 1758, -; 1676, +; 1675, +; 45, +)	TGACG(157, -)	----	Auxin: TGA-box (171, +), AuxRR(1881, +); GA: GARE(1573, -)
PG, Dil.10g001250.1	ABRE(1910, +; 1638, -; 565, +; 268, +; 257, +)	TGACG(413, -; 270, +)	TCA(1579, -)	GA: TATC-box(33, +), P-box(549, +; 178, +)
CX, Dil.09g022610.1	ABRE(1887, -; 1438, -)	CGTCA(1603, +; 1436, +; 497, +)	----	GA: TATC-box(1730, +)
β-Gal, Dil.09g014680.1	----	----	TCA(1819, -); SARE(1816, -)	----
NCED_Aril, Dil.14g000520.1	----	----	----	----
NCED_Pericarp, Dil.02g010420.1	ABRE(1793, -; 489, -; 487, -; 486, +; 431, +; 191, -; 190, -)	CGTCA(1852, +)	TCA(1814, +; 578, -)	Auxin: TGA-box(1855, -)
ABA2, Dil.10g020650.1	ABRE(174, -; 172, -; 166, +）	----	----	Auxin: TGA-element(1814, -)
AAO3, Dil.13g015420.1	----	----	TCA(175, -)	Auxin: TGA(669, -); GA: P-box(845, -)
ABF, Dil.09g011430.1	ABRE(850, +; 766, -; 642, +)	CGTCA(1946, +)	----	GA: GARE(581, -)

Table S3 (continued table) Prediction analysis of cis-elements related to hormone of promoter sequence in genes used in qRT-PCR.

Gene Name<br>Genome_ID	cis-elements related to hormone (Position, Strand)			
	**ABA**	**MeJA**	**SA**	**Others**
LOX2_Aril, Dil.13g013000.1	ABRE(222,-; 221, +)	----	----	Auxin: AuxRR(252, +);<br>GA: TATC(529, +)
LOX2_Pericarp, Dil.03g001200	ABRE(242, +)	TGACG(1490, +; 88, -)	TCA(1933, -)	
JAZ, Dil.01g002090.1	ABRE(1976, +; 1680, +; 1679, +; 389, +; 344, -; 342, -; 315, -; 314, +)	TGACG(1683, -; 1588, -; 1529, +; 1000, -)		GA: P-box(845, -);
PAL1_Aril, Dil.14g007600.1	ABRE(1854, -; 1758, +)	----	----	Auxin: TGA(1947, +);<br>GA: P-box(212, +)
PAL1_Pericarp, Dil.04g021490.1	ABRE(484, -; 482, -; 287, -; 286, +)	TGACG(422, +)	----	Auxin: TGA(1476, -; 459, -)<br>GA: P-box(1786, +)
PR-1, Dil.11g016370.1	ABRE(618, +; 395, +)	TGACG(1959, +; 586, -)	----	Auxin: TGA(728, +)
NPR3, Dil.07g000580.1	ABRE(118, -)	TGACG(250, +; 194, -)	----	Auxin: TGA(535, -; 185, +; 168, +);<br>GA: TATC(1225, +)

Figure S1 Changes of appearance and aril breakdown of 'Shixia' and 'Luosanmu' fruits during room-temperature storage.

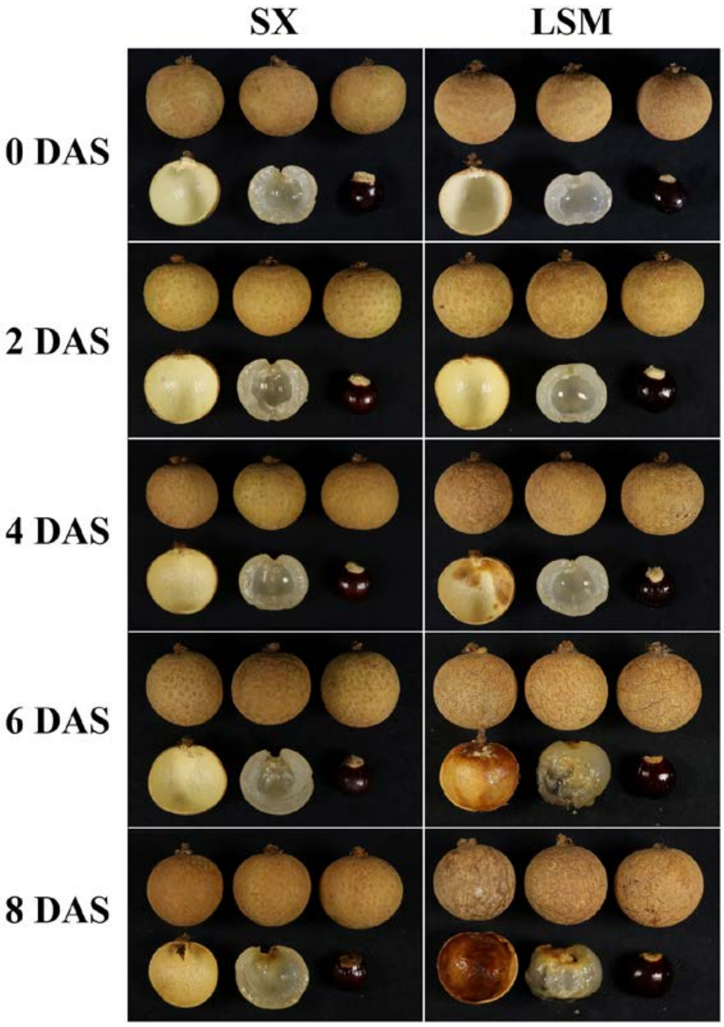

Supplement: Supplementary file 1 [file plants-11-02503-s001.zip › plants-1881521-supplementary.pdf]
